# Critical periods in *Drosophila* neural network development: Importance to network tuning and therapeutic potential

**DOI:** 10.3389/fphys.2022.1073307

**Published:** 2022-12-02

**Authors:** Bramwell Coulson, Iain Hunter, Sarah Doran, Jill Parkin, Matthias Landgraf, Richard A. Baines

**Affiliations:** ^1^ Division of Neuroscience, School of Biological Sciences, Faculty of Biology, Medicine and Health, University of Manchester, Manchester Academic Health Science Centre, Manchester, United Kingdom; ^2^ Department of Zoology, University of Cambridge, Cambridge, United Kingdom

**Keywords:** critical period, development, *Drosophila*, neuron, network, homeostasis

## Abstract

Critical periods are phases of heightened plasticity that occur during the development of neural networks. Beginning with pioneering work of Hubel and Wiesel, which identified a critical period for the formation of ocular dominance in mammalian visual network connectivity, critical periods have been identified for many circuits, both sensory and motor, and across phyla, suggesting a universal phenomenon. However, a key unanswered question remains why these forms of plasticity are restricted to specific developmental periods rather than being continuously present. The consequence of this temporal restriction is that activity perturbations during critical periods can have lasting and significant functional consequences for mature neural networks. From a developmental perspective, critical period plasticity might enable reproducibly robust network function to emerge from ensembles of cells, whose properties are necessarily variable and fluctuating. Critical periods also offer significant clinical opportunity. Imposed activity perturbation during these periods has shown remarkable beneficial outcomes in a range of animal models of neurological disease including epilepsy. In this review, we spotlight the recent identification of a locomotor critical period in *Drosophila* larva and describe how studying this model organism, because of its simplified nervous system and an almost complete wired connectome, offers an attractive prospect of understanding how activity during a critical period impacts a neuronal network.

## Introduction

Critical periods (CPs) are so-called because abnormal activity during such periods can induce permanent structural and/or functional change to a neuronal network. By contrast, the same manipulations prior to, or following, such periods have considerably reduced effects. First identified in the mammalian visual system (Hubel and Wiesel, 1970; Hubel et al., 1977), perhaps the best documented arise from studies which show that monocular deprivation, during a defined CP, is sufficient to skew the development of ocular dominance to favor the open eye; an effect that is greatly reduced when visual manipulation occurs after CP closure (Fagiolini and Hensch, 2000; Hensch, 2005; Morishita and Hensch, 2008; Gomez-Diaz et al., 2018; Hensch and Quinlan, 2018). That CPs exist, however, present something of a paradox. This is because it is established that experience-dependent plasticity is key to ensure optimal network configurations form. As such the restriction of heightened plasticity to time-limited defined windows is not, it would appear, supportive of future significant alterations to circuit function (Takesian and Hensch, 2013). This apparent conflict of early plasticity windows which enable optimization of circuitry versus the ensuing subsequent limitations to further significant adjustment is currently difficult to reconcile and will, undoubtedly, benefit from additional model systems, more amenable to experimental intervention.

**FIGURE 1:**
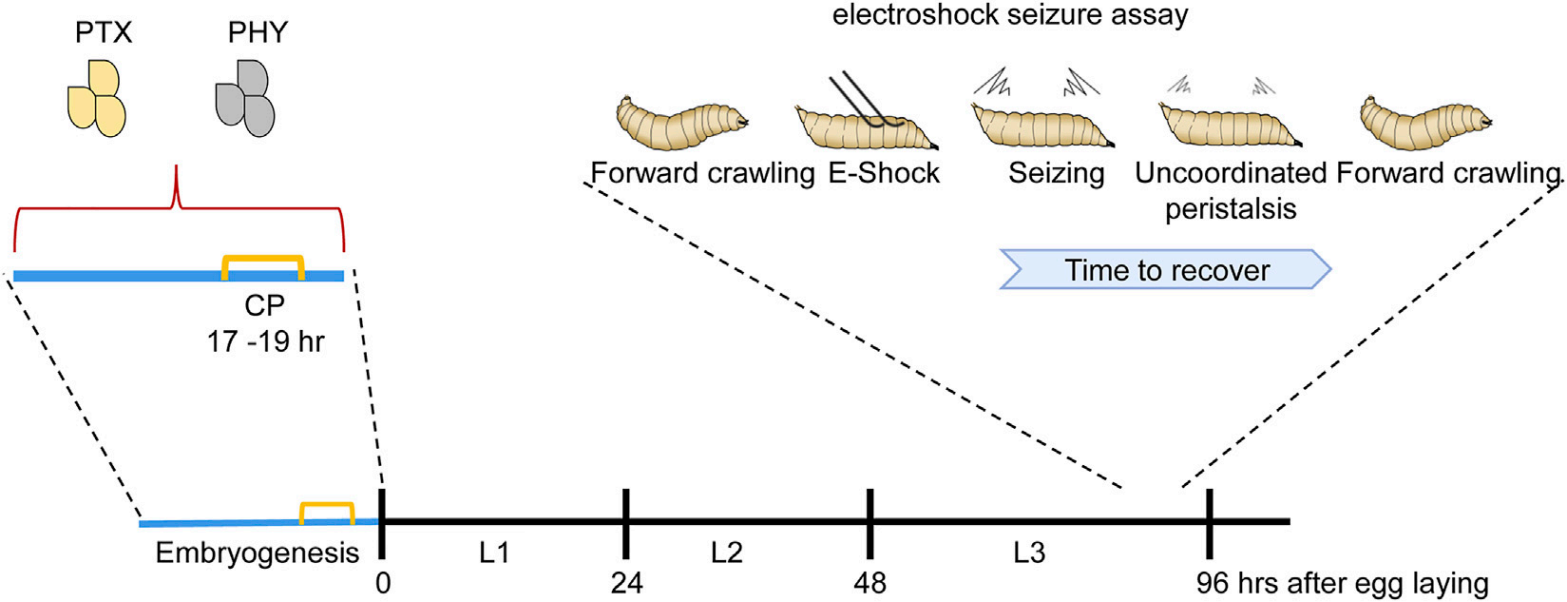
Manipulation of activity during the *Drosophila* locomotor CP is sufficient to induce a seizure-like phenotype. Manipulating activity during embryogenesis, in this instance *via* feeding gravid females either picrotoxin (PTX, excitatory) or phenytoin (PHY, inhibitory), is sufficient to disturb neuronal activity during the embryonic CP (indicated in yellow). Activity manipulation during this CP results in an unstable locomotor network. Thus, in response to electroshock, wall-climbing third instar larvae (L3), 5 days later and when no trace of drug is present, exhibit an extended seizure-like bout [for more details of this behavior see (Marley and Baines, 2011)]. L1 and L2 are first and second instar stages, respectively.

Developing circuits exhibit intrinsic activity. Where such activity has been visualized in detail, early spontaneous activity often transforms to patterned activity as a network matures. A good example is provided by the zebrafish spinal cord where calcium-imaging shows a transition from spontaneous irregular to patterned activity as development proceeds (Warp et al., 2012). A similar transition occurs in zebrafish optic tectum where pairwise correlations show increasing complexity, peaking at 5 days post fertilization (dpf). Moreover, activity-manipulation of visual experience, during these early stages in fish development, reduces hunting (a visually guided behavior) in older animals tested up to 9 dpf (Avitan et al., 2017). A similar transition of spontaneous to coordinated activity is evident in the development of the *Drosophila* larval locomotor circuitry. Early activity of body-wall muscles manifests as spontaneous twitch-like contractions restricted to individual segments. There is a notable transition as development proceeds, over the space of just a few hours, to patterned coordinated body muscle contractions that travel the length of body and which reflect mature larval peristalsis (Baines and Bate, 1998; Crisp et al., 2008; Crisp et al., 2011). Activity manipulations, *via* drug exposure, optogenetics or genetic manipulation, of the developing locomotor circuitry within the CNS have defined a CP that spans these two phases of activity (Giachello and Baines, 2015). As might be predicted, activity perturbation during this CP results in marked alteration to locomotor circuits in later larvae: specifically altered synaptic excitation of motoneurons and, at the whole network level, a heightened seizure-like activity in response to strong stimulation, in this example an electroshock (Giachello and Baines, 2015) (Figure 1).

In contrast to sensory networks, a requirement for activity to shape the development of a motor circuit is not immediately apparent. Indeed, the relative simplicity of motor circuits might be considered sufficient to allow intrinsic genetic programs to orchestrate development. A seemingly ubiquitous requirement for CPs, regardless of functional modality, is a strong argument against such a view and raises a fundamental question of which network parameters are influenced during such periods. Essentially, what is the role of a CP? That synaptic connectivity and behavior are influenced, in *Drosophila*, by developmental temperature suggests that such periods may allow developing nervous systems to compensate for unforeseen changes in the external environment (Kiral et al., 2021). Developing mammals, presumably more protected from changing temperature, nevertheless must compensate for stochastic influences due to genetic background, exposure to pathogens and/or dietary toxins. The existing models currently used to understand CPs, specifically mammalian sensory circuits, are complex which hampers understanding of how activity influences network tuning through modification of individual cellular excitable properties and synaptic connectivity. Thus, many questions remain unresolved; notably how plasticity mechanisms operating at the level of individual cells combine to achieve adjustment at the level of the network; and how early adjustment rules might differ, potentially fundamentally, from subsequent plasticity mechanisms.

### The *Drosophila* locomotor system offers experimental opportunity to understand critical periods

**FIGURE 2:**
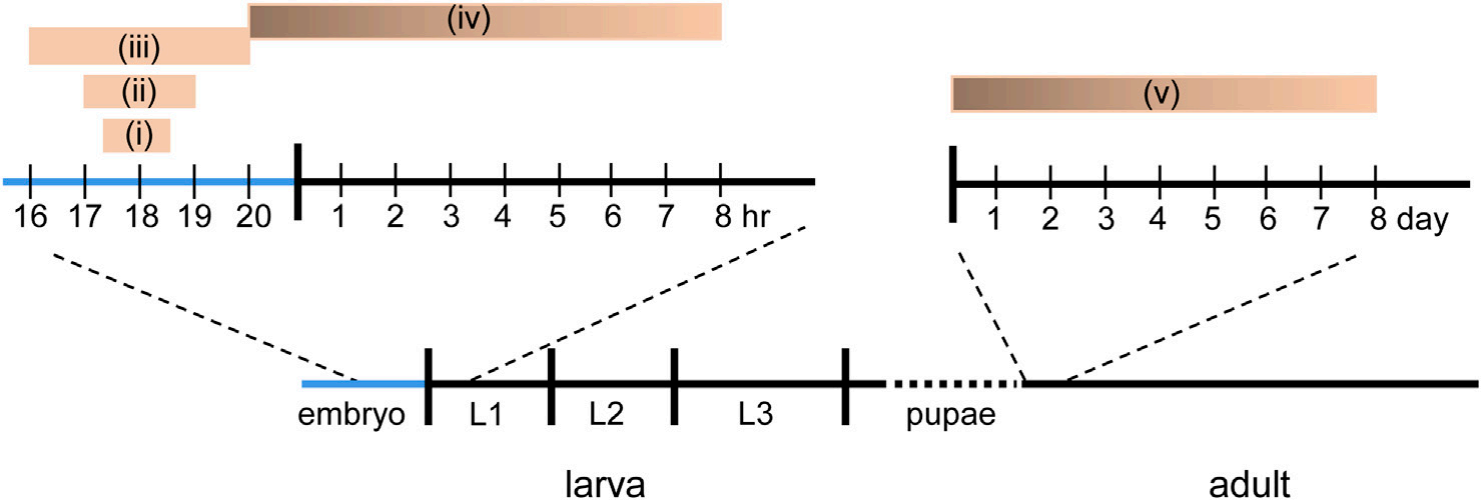
Known CPs in *Drosophila*. Five different, but in some cases, overlapping CPs have been reported in *Drosophila*. (i) Manipulation of activity between 17.5 and 18.5 h after egg laying (AEL) is sufficient to delay the development of the locomotor circuit to support hatching (Crisp et al., 2011). (ii) Manipulation of activity between 17–19 h AEL is sufficient to induce a seizure phenotype in larvae (Giachello and Baines, 2015). (iii) Manipulation of sensory input, *via* chordotonal neurons, between 16–20 h AEL is sufficient to reduce crawling speed in larva (Fushiki et al., 2013). (iv) Activity manipulation, in the last hr of embryogenesis through, with weakening influence, to 8 h after hatching, is sufficient to alter motoneuron dendritic growth (Ackerman et al., 2021). (v) Exposure to odor is sufficient to alter synaptic connectivity in the olfactory system within the first 2 days after eclosion (Golovin and Broadie, 2016; Golovin et al., 2019).

*Drosophila* has proven a powerful laboratory workhorse for both the identification of genes and mechanisms that orchestrate the development and function of the nervous system. In this regard it is notable that multiple CPs have been identified in the development of the *Drosophila* larval motor and adult olfactory circuits (Crisp et al., 2008; Crisp et al., 2011; Fushiki et al., 2013; Giachello and Baines, 2015; Giachello and Baines, 2017; Golovin et al., 2019; Ackerman et al., 2021) (Figure 2). The *Drosophila* larval motor network, in particular, offers the possibility to integrate both cell-specific and network effects of activity perturbation. This is because the larval *Drosophila* connectome is nearing completion, describing at the level of single cell identity, the “wired” connectivity map of the larval CNS (Ohyama et al., 2015; Fushiki et al., 2016; Zwart et al., 2016; Kohsaka et al., 2019; Zarin et al., 2019). Moreover, for locomotor circuits, normal neuronal function and deviations from the normal state are comparatively straightforward to determine, as usually explicit in the execution of behavioral (locomotor) outputs.

**FIGURE 3:**
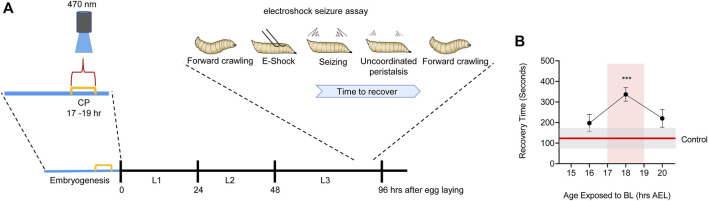
Manipulation of activity during *Drosophila* embryogenesis identifies a locomotor CP. **(A)** Activation of Cryptochrome-expressing neurons, by exposure to blue light (BL, 470 nm), can be exploited to increase activity in the CNS (Marley et al., 2014). Timed BL exposure localizes the embryonic locomotor CP to ∼17–19 h AEL (at 25°C). **(B)** Timeline for blue light exposure during embryogenesis and effect to seizure-like activity in subsequent wall-climbing third instar larvae. Only exposure to blue light during 17–19 h AEL is effective in inducing an increased seizure response. Blue light exposure before, or following, this 2 h window is less effective. For more details see (Giachello and Baines, 2015).

The *Drosophila* larval locomotor circuit comprises ∼33 identified motoneurons per hemi-segment that innervate ∼30 identified and accessible body wall muscles (Sink and Whitington, 1991; Landgraf et al., 1997; Hoang and Chiba, 2001; Landgraf et al., 2003; Zarin et al., 2019). Motoneurons receive excitatory synaptic drive from cholinergic premotor interneurons that form part of a central pattern generator (CPG) which is sufficient, without need for sensory input, to generate coordinated locomotor output (Suster and Bate, 2002). Electrophysiological recordings from motoneurons, *in vivo*, show that ion channels first express at around 13 h after egg laying (AEL): full embryogenesis taking ∼21 h (Baines and Bate, 1998). The first appearance of synaptic currents, an indicator of emergence of network function, occurs at 17 h AEL. Optogenetic manipulation of central neuron activity identified a 2 h period, beginning at 17 h AEL, which exhibits heightened sensitivity to activity perturbation (Giachello and Baines, 2015). Manipulating activity of interneurons during this period is sufficient to cause lasting changes that persist to the end of larval life. These changes lead to significantly lengthening of the recovery time to electroshock in mature larvae (tested 5 days post-manipulation). By contrast, manipulations before or after this 2 h window are relatively ineffective in leading to lasting change (Figure 3). We interpret this difference to mean that larvae manipulated during the CP, and that exhibit longer seizures as larvae, carry forward significant, and permanent, change to their locomotor networks which manifest as being less able to counter strong stimulation (i.e., that induced by an electroshock). Interestingly, the timing of this embryonic CP (17–19 h AEL) coincides precisely with the emergence of network function *via* synaptic transmission and the emergence of patterned activity in the CNS, leading to full peristaltic body wall muscle contractions (Baines and Bate, 1998; Crisp et al., 2008).

Genetic manipulation of central synaptic release shows that appropriately patterned network activity is necessary for coordinated locomotor movements to emerge on time. This is similarly indicative of this phase being essential for network tuning. By contrast, similar transient manipulations after onset of patterned activity are ineffective in delaying motor network maturation (Crisp et al., 2011). Two more recent studies add more to the understanding of this key stage of embryonic development. In the first, Carreira-Rosario et al. (2021) used calcium-imaging to monitor the embryonic progression of spontaneous muscle activity. They report that the transition from un-patterned to patterned muscular activity is key to the subsequent emergence of rhythmic locomotor CPG activity. Blocking muscular activity during these key stages resulted in larvae with altered behavior, which spent longer crawling rather than turning and/or head-casting (Carreira-Rosario et al., 2021). In the second study, Zeng et al. (2021), validated the key requirement for mechanosensory input to shape the developing locomotor CPG, and also identified what might be considered a “hub neuron” (the M-neuron), which is seemingly one of the first neurons to become active and needed for the development of the CPG (Zeng et al., 2021).

One of the unique strengths of the *Drosophila* locomotor network is that individual motoneurons are identifiable and accessible to patch electrodes: thus the “same” cell can be recorded across preparations and genotypes. Recordings from the anterior corner cell (aCC, MN1b) motoneuron show a profound change in synaptic drive following activity perturbation during the CP (Giachello and Baines, 2015). Specifically, excitatory cholinergic synaptic currents are increased in duration and drive greater action potential firing in aCC. It is particularly noteworthy that this change results following either imposed increased excitation (channelrhodpsin) or increased inhibition (halorhodpsin) during the CP, indicative that change from a pre-determined activity level is deterministic and not the direction of that change. The same change to excitatory synaptic currents is also seen in single gene mutations that alter neuronal activity (e.g., *para* ^*bss*^: an overactive hypermorph of the sole voltage-gated Na^+^ channel expressed in flies) or following exposure of the developing embryo to proconvulsants such as picrotoxin (PTX, a GABA_A_ receptor blocker) (Giachello and Baines, 2015). This consistency of outcome, regardless of the cause of activity perturbation, hints that developing networks utilize defined, activity-dependent, rules to tune intrinsic excitability and/or connectivity to maintain fixed outputs. If so, then it is tempting to speculate that such “tuning” rules are first enacted during a CP.

### Closure of a *Drosophila* critical period

In a recent study, Ackerman et al. (2021), reported a CP in the activity-dependent growth of motoneuron dendrites in the developing larval locomotor network. Focusing on well characterized motoneurons (specifically the aCC and RP2 cells), these authors showed that blockade of activity, during the last hr of embryogenesis (i.e., 20 h AEL), is sufficient to increase dendritic arbor growth, an effect that persisted, albeit weakening, through to 8 h after hatching. Potentiation of neuron activity resulted in an equivalent, but opposite effect, decreasing dendritic growth. Analysis of synaptic inputs, from identified premotor interneurons, showed that neuron silencing decreases inhibitory, but increases excitatory synapses on aCC/RP2. By contrast, potentiation of activity reduced excitatory synapse number but did not change number of inhibitory synapses. Similar effects were first reported in this same system following manipulation of excitatory cholinergic signaling. Blocking synthesis or release of acetylcholine (the principle excitor in the insect CNS) resulted in increased dendritic growth in aCC, whilst increasing the density of presynaptic release sites for this transmitter was sufficient to reduce dendritic growth (Tripodi et al., 2008). These changes appear compensatory and are indicative of an attempt by the locomotor network to maintain an appropriate excitation-inhibition balance. Moreover, activity manipulation during the earlier 17–19 h CP also significantly influences the excitation:inhibition balance, in this instance by favoring excitatory synaptic signaling (Giachello and Baines, 2015). After 8 h of larval life, activity manipulation fails to influence dendritic growth (Ackerman et al., 2021). The apparent closure of this CP coincides with astrocyte infiltration of the neuropil (Stork et al., 2014). Remarkably, genetic ablation of astrocytes extended the effect of activity manipulation on dendritic growth, an effect Ackerman et al. (2021), link to Neuroligin-Neurexin signaling. Closure of mammalian CPs, for example, in visual system, is also mediated by maturation of astrocytes, an effect linked with increasing expression of Connexin30 (Cx30) in these cells. Moreover, knockdown of Cx30 extended the CP in mouse visual cortex further indicative that maturation of astrocytes contributes to CP closure (Ribot et al., 2021). Astrocyte maturation which, together with components derived from neurons and oligodendrocytes, orchestrate the formation of perineuronal nets (PNNs). The assembly of these protein-based nets is activity-dependent, with their formation around neuronal soma demarking the beginning of CP closure. Thus, PNNs are widely believed to act as “plasticity brakes” [for review see (Takesian and Hensch, 2013; Gibel-Russo et al., 2022)].

### The *Drosophila* olfactory system exhibits a critical period

In addition to the larval motor system, the adult *Drosophila* olfactory system represents a similarly powerful model, because of a developing sensory network that is equally well characterized (Gomez-Diaz et al., 2018). Olfactory sensory neurons (OSNs) that express the same olfactory receptors (ORs) project to common glomeruli in a region of the central brain called the antennal lobes. This neuronal map had been considered hard-wired because loss of olfaction resulted in no obvious changes to connectivity (Larsson et al., 2004). However, more recent studies have shown that individual glomeruli can alter both their morphology and synaptic connectivity in response to manipulation of odors in early adult life [for a review see (Golovin and Broadie, 2016)]. For example, exposure of newly emerged adults (0–2 day old) to ethyl butyrate is sufficient to reduce innervation of the VM7 glomerulus by the Or42a OSN (expressing the receptor activated by this odorant). This reduction is due, in part, to an elimination of synaptic contacts that form between the OSN and VM7. Identical exposure at later stages, e.g., between 7 and 9 days after eclosion, has a much-reduced effect, consistent with the existence of a CP. This effect of precocious odorant exposure is blocked when activity of Or42a OSN was suppressed, indicative that afferent activity during a CP can drive tuning in a sensory network. Moreover, removing animals from ethyl butyrate exposure, whilst the CP is still open, results in most, but not all, animals showing a reversal of the remodeling that normally occurs in response to this odor indicating that, like mammalian sensory systems, errors made during a CP can become locked in (Golovin et al., 2019).

### Synaptic excitation is influenced following activity perturbation during a critical periods

The opening of a CP coincides with increasing GABA-mediated inhibition. Thus, manipulation of GABAergic signaling is sufficient to change CP onset, an effect that requires the presence of the GABA_A_ α1 receptor (Hensch et al., 1998; Fagiolini et al., 2004). The increase in inhibition is seemingly matched, at least where studied, by a fall in excitatory synaptic drive, again indicative of a change in the excitation:inhibition balance occurring during a CP (Zhang et al., 2018). This seeming anti-homeostatic effect (inhibition up and excitation down) has been observed, during a CP, in both visual and auditory cortex. These observations have led to the proposal of synaptic imbalance triggering, and characterizing, a CP [reviewed in (Wong-Riley, 2021)]. The locomotor CP in *Drosophila* also occurs at a time when GABAergic signaling is maturing (Kuppers et al., 2003) and, as we describe below, is similarly characterized by synaptic imbalance.

**FIGURE 4:**
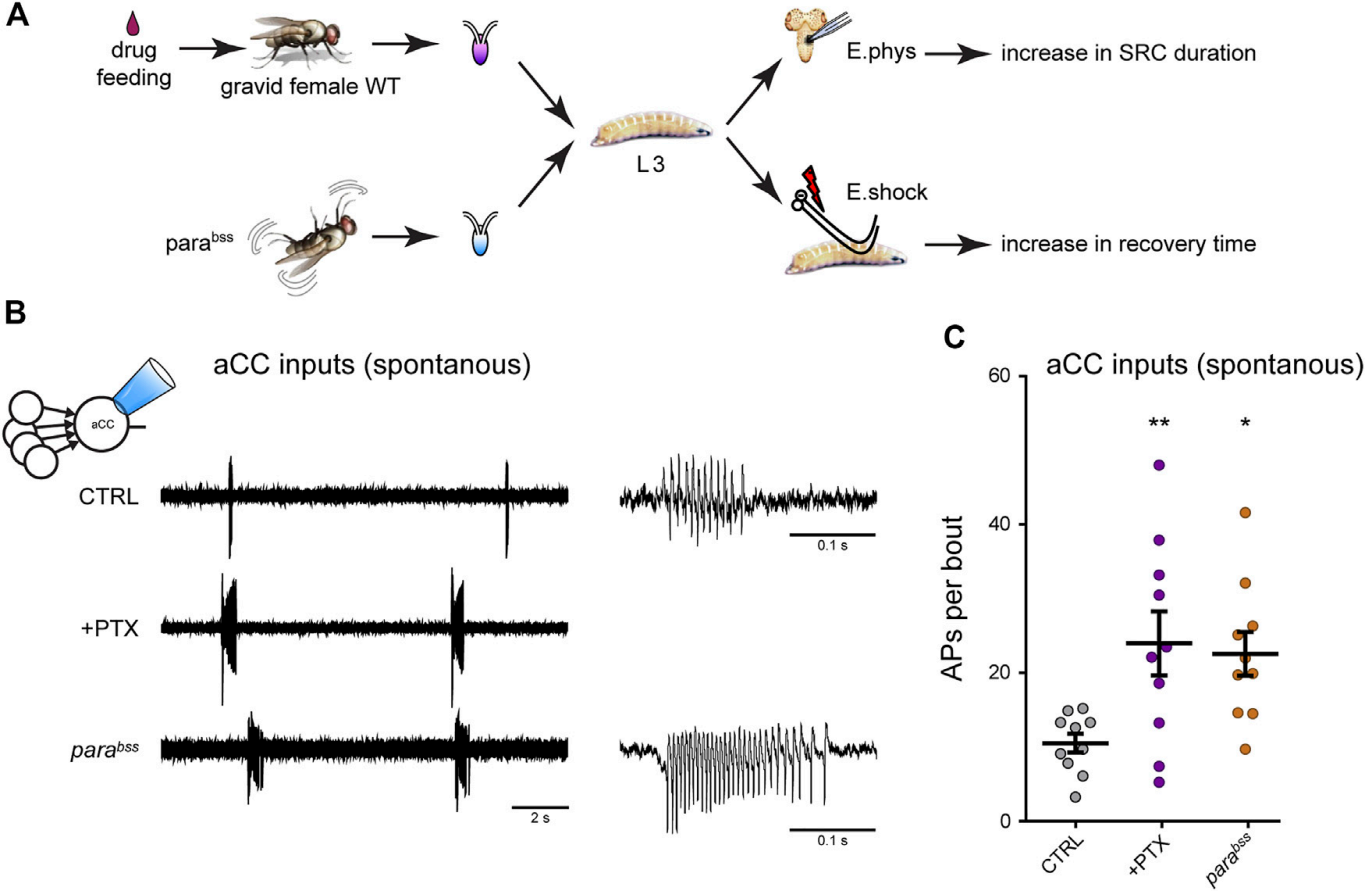
Manipulation of activity during the *Drosophila* locomotor CP results in a change to the excitation:inhibition balance **(A)** Exposure of gravid females to the proconvulsant compound PTX is sufficient to increase activity during the embryonic CP. The same is also achieved with the seizure-prone *para* ^*bss*^ mutant (Marley and Baines, 2011). Five days later, wall-climbing L3 larvae were tested by either electrophysiological recording from aCC motoneurons or by electroshock (E.shock), in order to measure change to synaptic drive (SRC, spontaneous rhythmic current), or susceptibility to induced seizure, respectively. **(B)** Recordings from L3-larval aCC motoneurons (*via* the loose-patch technique) show increased endogenous (i.e., spontaneous) spiking activity in conditions of excessive CP excitation (PTX and *para* ^*bss*^). The spontaneous activity is driven by the endogenous central locomotor CPG. **(C)** Quantification of the number of action potentials per bout. One-way ANOVA (F_(2, 27)_ = 3.91, *p* = 0.03) followed by Bonferroni’s post-hoc test (*n* = 10 in each group). This figure is reproduced from (Giachello et al., 2021) under a CC-BY license agreement.

The *Drosophila* larval connectome provides great promise for identifying those components of the larval CPG that are altered following activity perturbation during a CP. We have recently validated monosynaptic connectivity, that had been predicted by the connectome, for four identified premotor interneurons (INs) with the aCC motoneuron (Zarin et al., 2019; Giachello et al., 2022). These cells are the cholinergic excitors A27h and A18a, and the GABAergic inhibitors A23a and A31K. The identification of cell-selective “split” GAL4 driver lines [consisting of restricted expression of regulatory targets only in cells where the two components of the split-GAL4 activator are co-expressed (Luan et al., 2006)] for these cells allows their individual synaptic drive of motoneurons, elicited by optogenetic excitation, to be measured. Thus far, however, we have examined how the strength of all cholinergic (excitatory) synaptic drive was affected by activity manipulation during the embryonic locomotor CPG (Giachello and Baines, 2015). These measurements were carried out at the wandering third instar stage, some 5 days after CP perturbation. Thus, any changes observed would have been “locked in” by the transient embryonic CP perturbation. Our analysis showed that activity perturbation during the CP (achieved by exposing embryos to the proconvulsant PTX or use of the genetic seizure mutation, *para* ^*bss*^) results in increased excitatory synaptic drive not only during this period, but also throughout the larval life course, consistent with permanent change being locked-in (Marley and Baines, 2011). This change in excitatory synaptic drive was accompanied by increased action potential firing in aCC (Giachello et al., 2021) (Figure 4). Thus, our observations in *Drosophila* mirror, to some extent, those in mammals in that activity perturbation, during an embryonic CP, alters synaptic balance, both during the CP and, in this instance, beyond. However, studies in *Drosophila* are currently lacking to show how activity, in non-manipulated animals, changes during this CP.

### Nitric oxide transduces neural activity during a critical period

A foremost question is how changes between synaptic partners, that occur during a CP, may impact other cells and their connections within a network, either locally in a sub-network or more globally? Such changes may be in response to activity-driven plasticity (i.e., “activity-driven”) or may result from homeostatic change in response to plasticity (i.e., “network-driven”). Moreover, whilst it is considered that such changes will act to promote network stability and functional “robustness” (i.e., an ability to tolerate strong stimuli), the possibility remains that they may instead destabilize network activity to the point of producing aberrant outputs associated with neurological disease (see below). The relative simplicity, coupled to the known connectome, provided by the *Drosophila* larval locomotor network offers the prospect of addressing these fundamental issues.

A recent study identified nitric-oxide (NO) to mediate, at least in part, the effects of activity manipulation during the CP in the larval *Drosophila* motor network (Giachello et al., 2021). Thus, the effect of activity-perturbation, during this CP, is potentiated or blocked by simultaneous manipulation of this signaling pathway. Importantly, the effect of such manipulations are dependent on the basal activity state of the embryonic CNS. Thus, the effect of increased neural activity during the CP is prevented by blocking canonical NO-signaling. By contrast, reducing neural activity during the CP is overcome by increasing NO-signaling. These, and additional manipulations, again indicate that it is the level of activity, rather than activity *per se*, that determines network function *via*, at least in part, changes in NO production (Giachello et al., 2021). This seemingly duplicitous activity of NO has been highlighted before, in different systems, from cell survival (Calabrese et al., 2009) to nociceptive transmission (Jin et al., 2011) and, particularly, epileptogenesis where its role is highly contradictory with significant evidence supporting both proconvulsive, and anticonvulsive activity (Hrncic et al., 2012). Thus far, the diversity of the downstream target molecules of NO-signaling, coupled to the lack of homogeneity among different studies (differing drugs, dose and route of administration applied to diverse seizure models), make it difficult to identify a mechanism. Indeed, it has been reported that NO can differentially modulate excitatory (glutamatergic) and inhibitory (glycinergic) synapses in dorsal horn neurons (Jin et al., 2011). Thus, our recent results, which show that NO manipulation produces disparate effects dependent on the prior activity state of the pre-manipulated circuit, offers a potential explanation.

### Clinical relevance

Efforts to identify effective treatments for complex neurological disorders have largely been unsuccessful. Diseases such as epilepsy, autism and schizophrenia, remain significant burdens to society. A likely commonality between these diseases is they may derive from incorrect development of neural networks (Meredith et al., 2012; Marin, 2016; Ismail et al., 2017; Lee et al., 2017). Against this backdrop, the therapeutic potential of CPs is becoming evident. This is because recent studies show that activity manipulation, during such periods, may have significant clinical value by markedly reducing the impact of underlying causative mutations (Blumenfeld et al., 2008; Giachello and Baines, 2015; Marguet et al., 2015). Though this research is still very much in its infancy, we describe below some notable studies relating to epilepsy that serve to indicate the promise of CP manipulation.

Whilst progress continues in identifying genetic causes for epilepsy (Cunliffe et al., 2015; Noebels, 2015), the ability to manage seizures has remained frustratingly constant. Thus, despite the introduction of many new antiepileptic drugs (AEDs), roughly 30% of epilepsy sufferers remain refractory to drug treatment. Moreover, all AEDs are antiepileptic in that they reduce seizure frequency and/or severity but are not antiepileptogenic, i.e., they do not prevent the mechanism which leads to the occurrence of seizures in a brain. Thus, cessation of AED use invariably leads to recurrence of seizures. One of the first studies, showing that early treatment can have substantial benefit for epilepsy, exposed the absence-seizure model WAG/Rij rat to ethosuximide prior to first seizure onset. Pups fed ethosuximide, from p21 to 5 months, covering the period when seizures normally first manifest, showed few to no seizures up to age 8 months. The suppression of seizures was accompanied by changes in expression to ion channels (e.g., HCN, Na_v_1.1 and 1.6) that were consistent with seizure-suppression (Blumenfeld et al., 2008). Analysis beyond age 8 months has not, however, been reported. Thus, it cannot be stated whether this treatment merely delayed seizure onset or was, indeed, antiepileptogenic.

Manipulation of activity during the defined embryonic CP (17–19 h AEL) in the *Drosophila* locomotor circuit is equally sufficient to prevent the appearance of seizure-like activity in established single gene mutations used to model epilepsy. The first indication of this was provided by exposing developing embryos, carrying a mutation in the *slamdance* gene, to the AED phenytoin. Drug exposure was sufficient to prevent characteristic seizure-like activity in response to electroshock at third instar some 5 days later, when no traces of drug were present (Marley and Baines, 2011). The *slamdance* gene (recently renamed as *julius seizure*) encodes a membrane-spanning protein of unknown function but is a well-used model of seizure in *Drosophila* (Horne et al., 2017). A refinement of this experiment, using optogenetic stimulation of halorhodopsin to inhibit neuronal activity during embryogenesis, showed an identical outcome in the *para* ^*bss*^ seizure mutant (Giachello and Baines, 2015). Importantly, both the *sda* and *para* ^*bss*^ mutants show heightened synaptic excitation during embryogenesis that extends across the CP, consistent with aberrant activity during this period being sufficient to induce a seizure phenotype in larvae. As a test of this hypothesis, manipulating neuronal activity (either increasing or decreasing), in otherwise wild type embryos, is sufficient to induce seizure-like activity in larvae. Varying the time of optogenetic activity-manipulation again identified the 2 h period that is now defined as the locomotor CP (i.e., 17–19 h AEL). As expected, manipulated animals (seizure-rescued or seizure-induced) revert to their pre-manipulated genotypes during pupation to adult flies: a period when the nervous system is extensively rebuilt and when activity was not experimentally manipulated (Giachello and Baines, 2015). This remarkable finding has since been reproduced in mouse. Exposure of a K_v_7 mutant to bumetanide, during the first 2 weeks postnatal before seizures occur, is sufficient to prevent the subsequent emergence of epilepsy that occurs in littermates not exposed to drug (Marguet et al., 2015). Collectively, these experiments provide strong support for the hypothesis that manipulating activity during early neural development, especially during CPs, can counter the presence of destabilizing network activity patterns. This, in turn, supports the view that key signaling parameters, possibly including homeostatic setpoints, are set during these developmental windows.

Similar conclusions have been reached from studies of Fragile-X syndrome, schizophrenia and, notably, amblyopia (Hensch and Quinlan, 2018; Asiminas et al., 2019; Mukherjee et al., 2019). In the latter example, asymmetric development of visual circuits can result in a shift in ocular dominance to leave an affected eye reduced in visual acuity. This condition occurs during the first 8 years of age in children and is relatively easy to correct by favoring visual input to the affected eye. Treatment, however, becomes progressively less successful after this age. Our understanding of the role of CPs in the development of the mammalian visual system thus provides a more detailed mechanistic understanding for this treatment regime. For example, short duration monocular deprivation during the CP results in transient change to ocular dominance that fully reverses on restoration of normal vision (Schwarzkopf et al., 2007). By contrast, monocular deprivation that lasts beyond the CP produces a change to visual acuity in the deprived eye that is resistant to rescue on restoration of normal vision. Numerous studies, in both human and non-human primates, show that visual plasticity declines following the closure of the CP but, importantly, does not cease [reviewed in (Hensch and Quinlan, 2018)]. Thus, plasticity remains post-CP but, in the case of amblyopia, is slower and less pronounced. Whilst amblyopia is not directly relevant to *Drosophila*, the clear demonstration of being able to prevent a disorder by intervening during a CP is. Indeed, this further suggests that the CP identified in *Drosophila*, activity-manipulation of which prevents seizure in otherwise seizure-mutant backgrounds (Giachello and Baines, 2015), is likely to be highly similar to its mammalian counterparts.

## Conclusion

**FIGURE 5:**
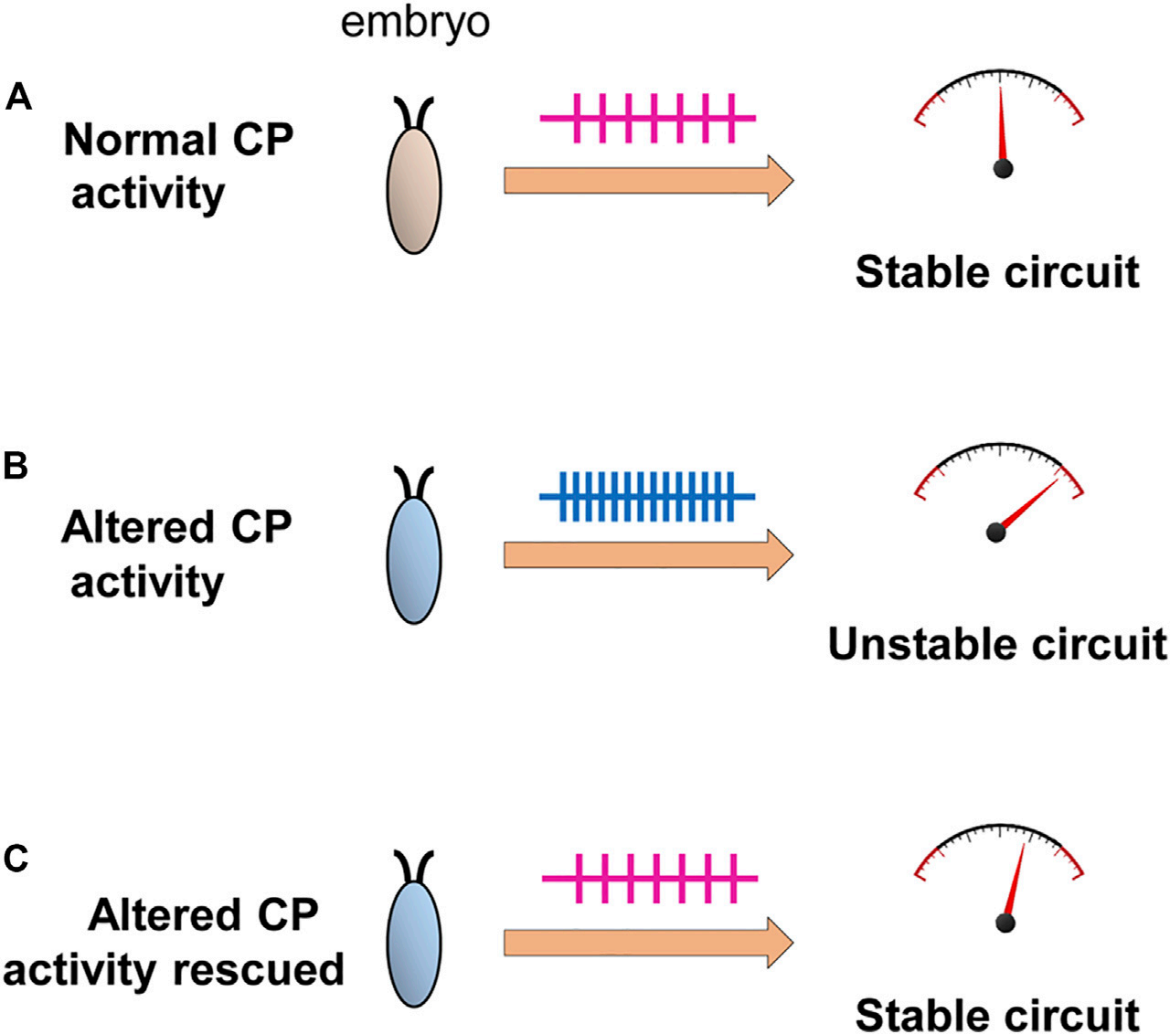
Activity-manipulation during the *Drosophila* locomotor CP alters network stability. Activity-manipulation during the locomotor CP is sufficient to permanently induce a seizure-like phenotype in later larvae. This is indicative of significant and permanent change to neuron/network stability. **(A)** Normal activity patterns during a CP, in a wildtype embryo, may allow the setting of physiologically appropriate homeostatic setpoint(s) that ensure robustness (i.e., an ability to compensate for strong stimuli) of the locomotor network. **(B)** Altered activity during the CP, by contrast, sets inappropriate setpoints(s) pushing the mature network closer to being less stable, particularly when subjected to a strong stimulus (i.e., an electroshock). **(C)** Rescue of activity during the CP, in backgrounds where activity is already perturbed, is sufficient to prevent the changes that would normally occur (Giachello and Baines, 2015) consistent with activity-rescue during this period forcing physiologically appropriate homeostatic setpoint(s) to be encoded.

Our understanding of the requirement for a CP in the development of a neural circuit remains incomplete. That such periods are evident across phyla and across sensory and motor circuits is indicative of a universal and critical role(s). Key to establish will be what “physiological parameters” are influenced by activity during a CP. An attractive hypothesis, that we support, posits that homeostatic set-points, which govern neuron and network excitability, are set during a CP (Figure 5). This would, if validated experimentally, provide a plausible mechanistic understanding for why aberrant activity during a CP induces permanent change to neuron and/or network function. Moreover, it would additionally explain why subsequent plasticity mechanisms are generally unable to “correct” the mis-adjustments induced during a CP (because an incorrectly specified set-point would constitute the “gravitational pull”). The identification of CPs in simpler organisms, such as *Drosophila*, offer the prospect of being able to experimentally address these, and other, questions with a greatly increased level of resolution.

The translational potential for increased understanding of CPs is significant. A paradox currently exists when considering manipulating a CP for clinical benefit: the onset of disease symptoms almost certainly occurs after the prime opportunity to intervene (i.e., the CP closing) has passed. In the absence of suitable biomarkers, reopening a CP may be required for clinical intervention. A few intriguing studies show that exposure to the AED, valproate, can reopen a CP (Silingardi et al., 2010; Gervain et al., 2013) most likely though its activity as a histone-deactylase inhibitor (Baroncelli et al., 2016). Similarly, reintroduction of immature astrocytes and/or degradation of PNNs have potential to extend or reopen CPs (Carulli and Verhaagen, 2021; Ribot et al., 2021). Thus, opportunities to reopen CPs exist, at least in the laboratory, which hopefully may translate to the clinic. It is to be hoped that activity-manipulation, during a reopened CP, may obviate the consequences of any initial activity disturbance that resulted in altered network activity.
